# Effects of physical activity on the levels of remnant cholesterol: A population‐based study

**DOI:** 10.1111/jcmm.18062

**Published:** 2023-11-29

**Authors:** Jingfei Chen, Qin Luo, Yingjie Su, Jiangang Wang, Zhenfei Fang, Fei Luo

**Affiliations:** ^1^ Research Institute of Blood Lipid and Atherosclerosis, The Second Xiangya Hospital Central South University Changsha Hunan China; ^2^ Reproductive Medicine Center, Department of Obstetrics and Gynecology, The Second Xiangya Hospital Central South University Changsha Hunan China; ^3^ Department of Cardiovascular Medicine, The Second Xiangya Hospital Central South University Changsha Hunan China; ^4^ Department of Emergency Medicine, The Affiliated Changsha Central Hospital, Hengyang Medical School University of South China Hengyang Hunan China; ^5^ Department of Health Management, The Third Xiangya Hospital Central South University Changsha Hunan China

**Keywords:** atherosclerotic cardiovascular disease, lipid accumulation, physical activity, remnant cholesterol

## Abstract

Physical activity (PA) has the potential to bring about favourable changes in plasma lipid profile. However, the relationship between PA and remnant cholesterol (RC) remains unclear. We aimed to study the link between PA and RC using the database of the 2007–2020 National Health and Nutrition Examination Survey (NHANES). PA was categorized based on Physical Activity Guidelines for Americans. A multivariate linear regression model was used to determine the correlations between PA and RC. The study involved a total of 18,396 participants and revealed that individuals whose PA met the guidelines by engaging in moderate‐intensity PA at least 150 min per week had lower body mass index and showed decreased levels of triglyceride, TC, and haemoglobin A1c compared to those who were physically inactive, exercising <150 min per week. Participants whose intensity of PA meets PA guidelines had a lower level of RC than those who did not met PA guidelines (*β* = −1.3, 95% confidence interval [CI]: −1.9 to −0.7, *p* < 0.001), even after adjusting for confounders. During subgroup analysis, we observed that race (*p*_interaction_ = 0.0089) emerged as a significant factor of interaction.

## 1 BACKGROUND

Atherosclerotic cardiovascular disease (ASCVD) continues to be one of the primary causes of mortality and disability worldwide. The causal risk factors for ASCVD have long been thought to include aberrant lipid metabolism, specifically increased low‐density lipoprotein cholesterol (LDL‐C) and triglycerides (TG). Lipid‐lowering drugs like statins are the first line and cornerstone drugs for the prevention and treatment of ASCVD.^1^, ^2^ Although statins can effectively reduce cholesterol levels, their ability to lower TG remains insufficient. In addition, even with intensive statin treatment, some individuals are unable to reach their LDL‐C goals, have intolerance to statins, or have considerable residual risk for cardiovascular disease. This encourages researchers to investigate novel lipid‐lowering strategies. Clinical practice recommends individuals with ASCVD reach an LDL‐C goal. However, the value of remnant cholesterol (RC) is underestimated. RC is defined by the amount of cholesterol present in triglyceride‐rich lipoproteins. RC consists of the cholesterol carried by very low‐density lipoprotein (VLDL) and VLDL remnants when in the fasting state, as well as that carried by chylomicron remnants in the non‐fasting state. RC has been established in an increasing number of studies to play a pivotal role in the development of ASCVD and contribute to the residual risk for cardiovascular disease. According to recent findings, RC, like VLDL‐C, may be responsible for about half of the risk of APOB‐associated myocardial infarction.^3^ In older persons at high cardiovascular risk, RC levels are related to major adverse cardiovascular events (MACE), irrespective of other risk variables.^4^ In recent years, RC has gained more attention as a causative risk factor for ASCVD and a potential new opportunity for reducing residual cardiovascular risk.^5^, ^6^

Physical activity (PA) is crucial to health regulation since it is significantly linked to obesity and metabolic disorders. According to findings from earlier studies, PA has the potential to aid in the treatment and prevention of ASCVD^7^, ^8^ and bring favourable changes in plasma lipid levels.^9^ A rise in both total cholesterol (TC) and LDL‐C was associated with prolonged periods of physical inactivity.^10^ However, there has been little investigation into the possible link between PA and RC. The major purpose of this study is to investigate the correlation between PA and RC as well as look for any relevant interacting elements that could be at play.

## 2 METHODS

### 2.1 Study design and data sources

The National Health and Nutrition Examination Survey (NHANES) is a nationwide survey conducted by the NCHS under the CDC since 1999. Interviews and physical exams were conducted at participants' homes utilizing mobile examination facilities to obtain data. Information, techniques, and resources are available to other researchers for replication or duplication of the study. The research design and data of NHANES can be accessed at https://www.cdc.gov/nchs/nhanes/.

### 2.2 Study population

Seven cycles of the NHANES were evaluated, covering the years from 2007 to 2020. The NHANES employs a stratified, multistage probability sampling method to ensure that the obtained sample accurately represents the entire population of the United States. The data include physical examinations, in‐home interviews, and laboratory testing conducted at Mobile Examination Centers.

### 2.3 PA Questionnaire

The measurement of PA relied on self‐reporting. The Global Physical Activity Questionnaire (GPAQ) inquired about the frequency and duration of PA in the realms of occupation, transportation and leisure‐time during a typical week. Transportation‐related PA refers to activities that involve walking or cycling. Occupation‐related PA encompasses the physical activities individuals engage in as part of their work or professional commitments. This includes tasks such as studying, training, performing household chores, participating in agricultural activities like harvesting food or crops, fishing, hunting for sustenance and seeking employment opportunities. On the contrary, leisure‐time PA refers to physical activities that individuals willingly pursue without significant pressure or obligation, primarily for recreational purposes. These activities may include sports, fitness exercises, and entertainment‐related pursuits. Individuals' levels of occupation‐related PA, transportation‐related PA, and leisure‐time PA were measured by Global Physical Activity Questionnaire. The measurement of PA included the description of intensity (vigorous vs. moderate), frequency (per week) and duration (in minutes) of each specific type of PA performed during a typical week. Both occupation‐related PA and leisure‐time PA were reported, considering the intensity as either vigorous or moderate. The minutes of vigorous PA were doubled and combined with the minutes of moderate PA for both occupation‐related PA and leisure‐time PA, as validated.^11^ The overall quantity of PA was calculated by adding the PA performed during leisure time, while work, and during travel. The Physical Activity Guidelines (2018) recommended moderate‐intensity PA for 150 min per week, vigorous‐intensity PA for 75 min per week, or an equal mix.^12^ Participants with insufficient PA according to the 2018 Physical Activity Guidelines were classified as ‘physically inactive’, while those who did were ‘physically active’.^12^

### 2.4 Assessment of outcomes

Complete instructions for taking and analysing blood samples are available in the NHANES Laboratory/Medical Technologists Procedures Manuals. Individuals' peripheral blood was collected in the morning after they had fasted for at least 8 h. Enzymatic analysis was used to determine HDL‐C, total cholesterol and triglyceride levels. LDL‐C levels were calculated by Friedewald calculation.

### 2.5 Collection of other variables

Age, sex, education and race were collected. Lifestyle factors including smoking and alcohol intake were included. Individuals with smoking history were divided into non‐smokers, past smokers and current smokers. Information of daily alcohol intake (g/day) was also collected. Body mass index (BMI), systolic blood pressure (SBP), diastole pressure (DBP), fasting insulin, and fasting plasma glucose (FPG), gamma‐glutamyl transferase (GGT), alanine aminotransferase (ALT), aspartate aminotransferase (AST), albumin, creatinine, uric acid, calcium, hypersensitive‐c‐reactive‐protein (hsCRP) and haemoglobin A1c (HbA1c) were collected. The information on hypertension, diabetes and use of lipid‐lowering medications is also included.

### 2.6 Statistical analysis

The data were analysed using the R language (version 4.2.0, http://www.R‐project.org, The R Foundation) and the statistical tool Empower Stats (version 4.1, http://www.empowerstats.com, X&Y Solutions, Inc., Boston, MA), with a significance level of *p* = 0.05. The data analysis included the right amount of weighting so that the results could be generalized to the total US population. The data were presented as mean ± standard deviations for continuous variables with normal distributions, while for variables with skewed distributions, the data were displayed as medians ± quartiles. The significance of the differences between the groups was determined using one‐way anova and the Kruskal–Wallis H test. Categorical data were presented as frequencies and percentages, where the significance of differences between the groups was examined using the chi‐squared test. A multivariate linear regression model was used to investigate the relationships between PA and RC, according to STROBE statement,^13^ we simultaneously showed the results from unadjusted and adjusted analyses. Stratified linear regression models and likelihood ratio tests were used to do subgroup analyses and estimate changes and interactions between subgroups.

## 3 RESULTS

### 3.1 The process of participant selection

**FIGURE 1:**
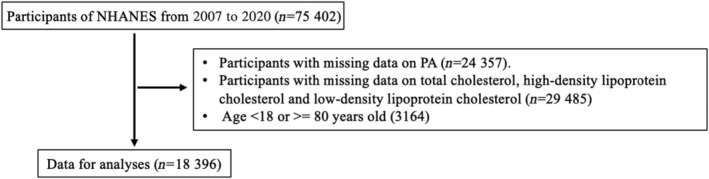
Flow chart for participants recruitment of this study, National Health and Nutrition Examination Survey 2007–2020.

After assessing the participants, 18,396 of 75,402 participants were included. 55,106 of the 75,402 participants were excluded from the analysis, of which 29,485 revealed no data on lipid profile, 24,357 showed missing data on PA, 3164 with age <18 or ≥80 years old (Figure 1).

### 3.2 Baseline characteristics of participants

aaaThe study included 18,396 participants (mean age: 46.8 ± 17.2 years, 48.2% men). All the included participants were classified as ‘physically active’ or ‘physically inactive’ according to total PA status regarding meeting the 2018 Physical Activity Guidelines. Baseline characteristics of participants are shown in Table 1. The study found that individuals in physically active group (>150 min per week) were more likely to be younger, male, and had a lower prevalence of diabetes and hypertension as compared to those who were physically inactive. Individuals in physically active group also have higher HDL‐C and lower BMI and lower levels of FPG, fasting insulin, HbA1c, ALT, TG, TC and hsCRP than those who were physically inactive.

**TABLE 1:** Characteristics of study participants based on total physical activity status.

Minutes per week	<150	≥150	*p*‐value
Age (years)	50.1 (49.5, 50.6)	43.4 (42.9, 43.9)	<0.0001
FPG (mmol/L)	6.1 (6.1, 6.2)	5.8 (5.8, 5.8)	<0.0001
Fasting insulin (pmol/L)	92.5 (89.6, 95.5)	73.5 (71.5, 75.6)	<0.0001
HbA1c	5.8 (5.7, 5.8)	5.5 (5.5, 5.6)	<0.0001
ALT (U/L)	24.0 (23.4, 24.5)	25.0 (24.5, 25.4)	0.0063
AST (U/L)	24.1 (23.6, 24.6)	24.6 (24.3, 24.9)	0.1513
Albumin (g/dL)	4.1 (4.1, 4.2)	4.2 (4.2, 4.3)	<0.0001
GGT (IU/L)	29.0 (28.1, 29.9)	26.5 (25.8, 27.2)	0.0001
Creatinine (μmol/L)	76.2 (74.9, 77.4)	76.3 (75.8, 76.8)	0.8473
Uric acid (μmol/L)	321.9 (318.8, 325.0)	323.8 (321.5, 326.1)	0.3414
Calcium (mmol/L)	2.3 (2.3, 2.3)	2.3 (2.3, 2.3)	0.0660
TG (mg/dL)	121.5 (118.9, 124.1)	109.3 (107.3, 111.3)	<0.0001
TC (mg/dL)	191.1 (189.5, 192.8)	189.1 (187.8, 190.4)	0.0372
HDL‐C (mg/dL)	53.5 (53.0, 54.1)	54.7 (54.2, 55.3)	0.0009
LDL‐C (mg/dL)	113.3 (111.9, 114.7)	112.5 (111.5, 113.6)	0.3926
hsCRP (mg/L)	4.5 (4.1, 4.9)	3.5 (3.2, 3.8)	0.0001
SBP (mmHg)	121.6 (120.9, 122.2)	119.4 (118.9, 119.8)	<0.0001
DBP (mmHg)	69.8 (69.4, 70.3)	70.2 (69.7, 70.7)	0.1089
BMI (kg/m^2^)	30.3 (30.1, 30.6)	28.5 (28.3, 28.7)	<0.0001
Alcohol (g/day)	8.0 (7.2, 8.8)	12.3 (11.5, 13.1)	<0.0001
Sex			
Male	37.7 (36.1, 39.2)	54.3 (53.1, 55.4)	<0.0001
Female	62.3 (60.8, 63.9)	45.7 (44.6, 46.9)	
Race			
Non‐Hispanic Black	12.5 (11.0, 14.2)	10.1 (8.9, 11.5)	<0.0001
Non‐Hispanic White	61.3 (58.4, 64.2)	66.9 (64.4, 69.2)	
Mexican American	9.7 (8.1, 11.6)	8.9 (7.7, 10.2)	
Other Hispanic	7.1 (5.9, 8.5)	6.0 (5.2, 7.0)	
Other Race	9.3 (8.3, 10.5)	8.1 (7.3, 9.1)	
Marital status			
Alone	36.2 (34.3, 38.1)	35.6 (33.8, 37.4)	0.5736
Live with partner	63.8 (61.9, 65.7)	64.4 (62.6, 66.2)	
Hypertension			
No	57.0 (55.2, 58.7)	69.7 (68.2, 71.1)	<0.0001
Yes	43.0 (41.3, 44.8)	30.3 (28.9, 31.8)	
Diabetes			
No	59.1 (57.0, 61.1)	72.9 (71.6, 74.3)	<0.0001
IFG	11.5 (10.2, 13.0)	10.6 (9.6, 11.6)	
IGT	7.2 (6.3, 8.2)	4.6 (4.1, 5.2)	
DM	22.2 (20.8, 23.6)	11.8 (11.1, 12.7)	
Drug_anti‐hyperlipidaemic			
No	75.8 (74.1, 77.3)	85.3 (84.3, 86.4)	<0.0001
Yes	24.2 (22.7, 25.9)	14.7 (13.6, 15.7)	

*Note*: Values are weighted mean (95% confidence interval) or weighted % (95% confidence interval). We categorized physical activity as physically inactive, who did not meet guideline and physically active who met guideline according to the 2018 Physical Activity Guidelines for Americans (adults engage in ≥150 min/week of moderate‐intensity activity per week, 75 min/week of vigorous‐intensity activity per week, or an equivalent combination).

Abbreviations: ALT, alanine aminotransferase; AST, aspartate transaminase; BMI, body mass index; DBP, diastolic pressure; DM, diabetes mellitus; FPG, fasting plasma glucose; GGT, γ‐glutamyl transpeptidase; HbA1C, haemoglobin A1c; HDL‐C, high‐density lipoprotein cholesterol; hsCRP, hs C‐reactive protein; IFG, impaired fasting glucose; IGT, impaired glucose tolerance; LDL‐C, low‐density lipoprotein cholesterol; SBP, systolic pressure; TC, total cholesterol; TG, triglyceride.

### 3.3 Univariate analysis

Table 2 shows the findings of the univariate analysis. The results revealed age, BMI, FPG, fasting insulin, HbA1c, ALT, AST, GGT, creatinine, uric acid, calcium, TG, TC, LDL, hsCRP, SBP, DBP, smoking status, hypertension and diabetes were positively correlated with RC. Levels of albumin and HDL‐C were negatively correlated with RC.

**TABLE 2:** The results of the univariate analysis.

Variables	*β* (95% CI)	*p*‐value
Age (years)	0.1 (0.1, 0.1)	<0.0001
Sex		
Male	Ref.	
Female	−3.0 (−3.5, −2.4)	<0.0001
Race		
Non‐Hispanic Black	Ref.	
Non‐Hispanic White	5.6 (5.0, 6.2)	<0.0001
Mexican American	7.1 (6.3, 7.9)	<0.0001
Other Hispanic	5.8 (4.9, 6.6)	<0.0001
Other Race Including Multi‐Racial	5.5 (4.6, 6.4)	<0.0001
Fasting plasma glucose	1.8 (1.6, 2.0)	<0.0001
Fasting insulin	0.0 (0.0, 0.0)	<0.0001
HbA1c	3.1 (2.7, 3.5)	<0.0001
ALT (U/L)	0.1 (0.1, 0.1)	<0.0001
AST (U/L)	0.0 (0.0, 0.1)	0.0001
Albumin (g/dL)	−1.1 (−2.0, −0.2)	0.0224
GGT (IU/L)	0.1 (0.0, 0.1)	<0.0001
Creatinine (μmol/L)	0.0 (0.0, 0.0)	<0.0001
Uric acid (μmol/L)	0.0 (0.0, 0.0)	<0.0001
Calcium (mmol/L)	9.8 (6.7, 12.9)	<0.0001
TG (mg/dL)	0.2 (0.2, 0.2)	<0.0001
TC (mg/dL)	0.1 (0.1, 0.1)	<0.0001
HDL (mg/dL)	−0.3 (−0.4, −0.3)	<0.0001
LDL (mg/dL)	0.1 (0.1, 0.1)	<0.0001
hsCRP (mg/L)	0.1 (0.1, 0.2)	<0.0001
SBP (mmHg)	0.1 (0.1, 0.2)	<0.0001
DBP (mmHg)	0.2 (0.2, 0.2)	<0.0001
BMI (kg/m^2^)	0.4 (0.4, 0.5)	<0.0001
Smoking status		
Never	Ref.	
Former	2.5 (1.7, 3.3)	<0.0001
Now	3.2 (2.4, 3.9)	<0.0001
Alcohol (g/day)	0.0 (−0.0, 0.0)	0.8751
Hypertension		
No	Ref.	
Yes	5.2 (4.5, 5.8)	<0.0001
Diabetes		
No	Ref.	
IFG	5.0 (4.1, 6.0)	<0.0001
IGT	5.9 (4.9, 7.0)	<0.0001
DM	8.3 (7.5, 9.2)	<0.0001
Drug_anti‐hyperlipidaemic		
No	Ref.	
Yes	4.3 (3.6, 5.1)	<0.0001

Abbreviations: ALT, alanine aminotransferase; AST, aspartate transaminase; BMI, body mass index; DBP, diastolic pressure; DM, diabetes mellitus; GGT, γ‐glutamyl transpeptidase; HbA1C, haemoglobin A1c; HDL‐C, high‐density lipoprotein cholesterol; hsCRP, hs C‐reactive protein; IFG, impaired fasting glucose; IGT, impaired glucose tolerance; LDL‐C, low‐density lipoprotein cholesterol; SBP, systolic pressure; TC, total cholesterol; TG, triglyceride.

### 3.4 The relationship between PA and RC

Table 3 presents the results of multivariate linear regression analyses conducted to investigate the association between total PA or different types of PA and the outcome variable, RC. In the crude model, participates who met the PA guideline had a lower RC than those who did not meet PA recommendations (*β* = −2.4, 95% CI: −3.0, −1.9, *p* < 0.001). After adjustment for age, sex, race and BMI in Model I, the relationship between PA and RC was still robust (*β* = −1.7, 95% CI: −2.2, −1.1, *p* < 0.001). In Model II, we adjusted for FPG, HbA1c, ALT, AST, SBP, CR, UA, smoking status and alcohol consumption on the basis of Model I, their association persisted (*β* = −1.4, 95% CI: −2.1, −0.8, *p* < 0.001). In fully adjusted model, the negative association remains significant (*β* = −1.3, 95% CI: −1.9, −0.7, *p* < 0.001). Regarding leisure‐time PA, comparable trends were observed. However, neither occupation‐related PA nor transportation‐related PA demonstrated any association with RC in either of the models. To conduct a sensitivity analysis and assess potential dose–response relationships between various types of PA and RC, we categorized PA into four groups: 0–149, 150–299 and > 300 min per week (Table 4). Similar inverse associations were observed across total amount and leisure‐time PA categories and RC levels, but no such associations were observed transportation‐related PA and occupation‐related PA with RC levels.

**TABLE 3:** Association between PA and RC in different models based on the PA guideline.

	Crude model		Model I	Model II		Model III		
	*β* (95% CI)	*p*	*β* (95% CI)	*p*	*β* (95% CI)	*p*	*β* (95% CI)	*p*
Total PA								
No	Ref.		Ref.		Ref.		Ref.	
Yes	−2.4 (−3.0, −1.9)	<0.001	−1.7 (−2.2, −1.1)	<0.001	−1.4 (−2.1, −0.8)	<0.001	−1.3 (−1.9, −0.7)	<0.001
Leisure‐time PA								
No	Ref.		Ref.		Ref.		Ref.	
Yes	−3.2 (−3.7, −2.6)	<0.001	−2.0 (−2.6, −1.5)	<0.001	−1.6 (−2.1, −1.0)	<0.001	−1.5 (−2.0, −0.9)	<0.001
Transportation‐related PA								
No	Ref.		Ref.		Ref.		Ref.	
Yes	−1.0 (−1.6, −0.4)	<0.001	0.2 (−0.4, 0.8)	0.554	0.1 (−0.6, 0.7)	0.0864	0.1 (−0.6, 0.7)	0.827
Occupation‐related PA								
No	Ref.		Ref.		Ref.		Ref.	
Yes	−0.2 (−0.7, 0.3)	0.103	−0.4 (−0.9, 0.1)	0.095	−0.4 (−1.0, 0.1)	0.139	−0.4 (−0.9, 0.2)	0.200

*Note*: No, not meeting PA guideline; Yes, meeting PA guideline. Model I adjusted for age, sex, race and BMI; Model II adjusted for model I plus FPG, HbA1c, ALT, AST, SBP, CR, UA, smoking status, and alcohol consumption. Model III adjusted for model I and II plus diabetes, hypertension, anti‐hyperlipidaemic drug.

Abbreviations: CI: confidence interval. PA_time: total physical activity time (hours per week).

**TABLE 4:** Association between PA and RC in different models based on the amount of PA.

	Crude model		Model I		Model II		Model III	
	*β* (95% CI)	*p*	*β* (95% CI)	*p*	*β* (95% CI)	*p*	*β* (95% CI)	*p*
Total PA time								
0	Ref.		Ref.		Ref.		Ref.	
1–149	−0.8 (−1.8, 0.2)	0.137	−0.2 (−1.2, 0.8)	0.651	−0.0 (−1.1, 1.0)	<0.001	0.1 (−0.9, 1.2)	0.811
150–299	−1.7 (−2.7, −0.7)	0.002	−1.0 (−2.0, 0.0)	0.061	−1.0 (−2.1, 0.0)	0.062	−0.9 (−1.9, 0.1)	0.086
≥300	−2.9 (−3.6, −2.3)	<0.001	−2.0 (−2.6, −1.3)	<0.001	−1.5 (−2.2, −0.8)	0.001	−1.3 (−2.1, −0.6)	0.001
*p*_trend_		<0.001		<0.001		<0.001		<0.001
Leisure‐time PA								
0	Ref.		Ref.		Ref.		Ref.	
1–149	−0.8 (−1.6, −0.1)	0.037	−0.3 (−1.1, 0.4)	0.408	0.1 (−0.7, 0.9)	0.850	0.2 (−0.6, 1.0)	0.687
150–299	−1.8 (−2.7, −0.9)	<0.001	−1.0 (−1.8, −0.2)	0.014	−1.0 (−1.8, −0.1)	0.038	−1.0 (−1.9, −0.1)	0.030
≥300	−4.2 (−4.8, −3.5)	<0.001	−2.7 (−3.3, −2.1)	<0.001	−1.9 (−2.6, −1.2)	<0.001	−1.6 (−2.3, −0.9)	<0.001
*p*_trend_		<0.001		<0.001		<0.001		<0.001
Transportation‐related PA								
0	Ref.		Ref.		Ref.		Ref.	
1–149	−1.0 (−1.8, −0.1)	0.030	0.1 (−0.7, 0.9)	0.793	0.4 (−0.4, 1.3)	0.343	0.3 (−0.5, 1.2)	0.457
150–299	−1.3 (−2.3, −0.3)	0.013	0.0 (−0.9, 0.9)	0.960	−0.1 (−1.2, 1.0)	0.912	−0.1 (−1.2, 1.0)	0.833
≥300	−1.0 (−1.9, −0.2)	0.021	0.3 (−0.5, 1.2)	0.454	0.2 (−0.6, 1.1)	0.575	0.3 (−0.6, 1.1)	0.515
*p*_trend_		0.001		0.486		0.537		0.554
Occupation‐related PA								
0	Ref.		Ref.		Ref.		Ref.	
1–149	−0.3 (−1.4, 0.7)	0.501	−0.7 (−1.6, 0.3)	0.189	−0.7 (−1.8, 0.4)	0.232	−0.5 (−1.6, 0.5)	0.332
150–299	0.6(−0.5, 1.8)	0.292	0.1(−1.1, 1.2)	0.928	0.1 (−1.2, 1.5)	0.833	0.0 (−1.4, 1.4)	0.991
≥300	−0.4 (−0.9, 0.1)	0.144	−0.6 (−1.1, −0.1)	0.014	−0.6 (−1.2, −0.1)	0.033	−0.5 (−1.1, 0.0)	0.069
*p*_trend_		0.234		0.025		0.049		0.095

### 3.5 The results of subgroup analyses

We used the likelihood ratio test to investigate the association between PA and RC and to compare results across subgroups stratification by sex, race, BMI, age, hypertension, diabetes and anti‐hyperlipidaemic medication use (Table 5). According to these findings, race is a potential confounding variable that might modify the association between PA and RC risk.

**TABLE 5:** Subgroup analysis for the association between PA and RC.

Variables	*β* (95% CI)	*p* value	*p*_interaction_
Age			
Below 60	−1.4 (−2.2, −0.7)	0.0003	0.7002
Over 60	−1.7 (−2.6, −0.7)	0.0010	
Sex			
Male	−1.2 (−2.4, −0.1)	0.0301	0.9068
Female	−1.3 (−2.1, −0.6)	0.0010	
BMI			
<18.5	−0.5 (−3.4, 2.4)	0.7482	0.7832
≥18.5, <24	−1.5 (−2.5, −0.5)	0.0038	
≥24	−1.3 (−2.1, −0.6)	0.0007	
Race			
Non‐Hispanic Black	−0.1 (−1.0, 0.9)	0.9130	0.0089
Non‐Hispanic White	−1.6 (−2.5, −0.7)	0.0013	
Mexican American	0.4 (−0.7, 1.6)	0.4786	
Other Hispanic	−1.6 (−2.9, −0.3)	0.0219	
Other Race	−2.2 (−4.1, −0.3)	0.0247	
Hypertension			
No	−1.3 (−2.0, −0.6)	0.0004	0.8543
Yes	−1.2 (−2.3, −0.1)	0.0305	
DM			
No	−1.1 (−1.8, −0.4)	0.0024	0.8558
IFG	−2.0 (−4.2, 0.3)	0.0886	
IGT	−1.9 (−3.7, 0.0)	0.0568	
DM	−1.2 (−2.8, 0.4)	0.1524	
Anti‐hyperlipidaemic drug			
No	−1.2 (−1.8, −0.5)	0.0006	0.5569
Yes	−1.7 (−3.5, 0.0)	0.0592	

*Note*: Above model adjusted for age, sex, BMI, race, FPG, HbA1C, ALT, AST, SBP, CR, UA, smoking status, alcohol consumption, diabetes, hypertension, anti‐hyperlipidaemic drug. In each case, the model is not adjusted for the stratification variable.

## 4 DISCUSSION

In this population‐based research of adults in the United States, we found individuals who were physically active had lower RC levels. Importantly, leisure‐time PA exhibits a dose‐dependent negative impact on RC, regardless of confounding factors. Studies have shown that PA has an inverse relationship with atherogenic lipid profiles.^9^ However, the connection between PA and RC is unclear. To the best of our knowledge, we are the first to provide evidence of a dose‐dependent relationship between PA and RC in a general population. In a subgroup study, the association between PA and RC was shown to vary depending on race. Our findings highlight the need to maintain and expand public initiatives to encourage regular PA practice, with the objective of improving the lipid profile of the population.

Despite abundant data showing a decrease in ASCVD risk with regular exercise,^8^, ^14^, ^15^, ^16^ the molecular processes responsible for this association are still unclear. Previous research has linked high PA with a healthier lipid profile, which may partially explain the beneficial effect of exercise on the cardiovascular system. LDLs are supposed to be atherogenic, while HDLs are thought to be antiatherogenic. Research reported that high levels of exercise were connected with lower triglyceride levels and higher HDL‐C levels, whereas inactivity or insufficient activity were linked to higher odds ratios for aberrant TG and HDL‐C.^9^, ^17^, ^18^ Apart from causing quantitative changes in blood lipids, exercise improves the quality of HDL particles.^19^ Studies have reported that both acute and chronic aerobic exercise can increase plasma HDL‐C levels in a dose–response manner.^19^ Several high‐quality meta‐analyses have also indicated that exercise can raise HDL‐C levels, with exercise volume exerting a greater impact on the HDL‐C response compared to exercise intensity.^20^, ^21^, ^22^ The current study similarly demonstrates that exercise can elevate HDL‐C levels. Numerous studies have shown that low levels of HDL‐C are associated with increased risk of cardiovascular disease. Therefore, for individuals with low HDL‐C levels, especially those with cardiovascular disease, exercise may provide cardiovascular benefits by raising HDL‐C levels. Furthermore, research suggests a close relationship between HDL cholesterol efflux capacity and cardiovascular health, and exercise has been shown to improve HDL cholesterol efflux capacity,^19^, ^23^ further suggesting that exercise may protect the cardiovascular system through HDL improvement. Our study provides further evidence of the positive effect of exercise on improving blood lipid profiles probably by regulating RC. RC refers to the cholesterol content of TRLs, which includes VLDL, IDL and chylomicron remnants.^24^ VLDL is produced by the liver while chylomicron remnants come from the intestines. Research reported that PA can improve hepatic lipid metabolism by reducing hepatic lipids,^25^, ^26^ indicating PA may reduce the production of VLDL. Free fatty acids (FFAs) from adipose tissue lipolysis and the hydrolysis of TG in VLDL will be burned off by exercise, which in turn increases the turnover of VLDL. Therefore, PA may reduce RC by downregulating the secretion of hepatic VLDL and increasing the turnover of VLDL. Koutsari et al. also found that daily aerobic exercise reduces fasting hypertriglyceridemia and postprandial lipaemia in healthy postmenopausal ladies.^25^ Thus, the low level of RC associated with exercise may be related to the protective effect of exercise on the cardiovascular system.

Physical activity (PA) is regarded as an essential component of lifestyle adjustment in the treatment of metabolic associated disease.^27^ The World Health Organization (WHO) recommends 75–150 min of high‐intensity PA per week, or 150–300 min of moderate to vigorous PA per week, for optimal health.^28^ Based on our findings, individuals with high PA are associated with lower RC, which may have a cardio‐protective effect. Based on our findings, clinicians can potentially advise adults who are PA inactive to increase their PA in accordance with the current guidelines. This recommendation aims to provide substantial benefits to the cardiovascular system. Besides, the current study also found a negative correlation between leisure‐time PA and RC, but there was no similar correlation observed with occupation‐related PA or transportation‐related PA. The reasons for these different results are still unclear and require further investigation. The outcomes of different types of PA vary, providing us with more opportunities for intervention. Previous studies have also shown that leisure‐time PA can improve lipid metabolism and have cardiovascular protective effects.^29^, ^30^, ^31^ Therefore, for patients with dyslipidaemia, clinicians may consider recommending appropriate increases in leisure‐time PA to improve lipid profiles and confer cardiovascular benefits.

The research that is currently being conducted does have certain limitations. Self‐reporting was used to determine the participant's activity level and to assess some of the risk factors. It is possible that a more precise examination of these factors may have resulted in a different contribution of PA to reduction in the levels of RC. Unfortunately, we were unable to demonstrate temporal causation for the observed correlation that we identified since the study was conducted using a cross‐sectional technique. Due to a lack of follow‐up information, we were unable to evaluate the dynamics of PA status and RC over time.

## 5 CONCLUSIONS

In conclusion, our data indicate a negative dose‐dependent connection between PA and RC. Despite the fact that there is a correlation between higher RC levels and an elevated risk of ASCVD, RC is still not a regularly utilized indicator and therapeutic target in clinical practice for lipid‐lowering. However, because of the critical role that RC plays in the development of ASCVD, it is important to give this factor greater consideration and to measure RC, particularly in the PA‐inactive population. Besides, these findings suggest that exploring the potential of increased PA as a strategy to reduce RC is warranted.

## AUTHOR CONTRIBUTIONS

**Jingfei Chen:** Conceptualization (lead); data curation (lead); formal analysis (lead); funding acquisition (lead); investigation (lead); methodology (lead); project administration (lead); writing – original draft (lead); writing – review and editing (lead). **Qin Luo:** Writing – original draft (equal). **Yingjie Su:** Writing – review and editing (equal). **Jiangang Wang:** Funding acquisition (equal); writing – review and editing (equal). **Zhenfei Fang:** Writing – review and editing (equal). **Fei Luo:** Conceptualization (lead); data curation (lead); formal analysis (lead); funding acquisition (lead); investigation (lead); methodology (lead); project administration (lead); writing – original draft (lead); writing – review and editing (lead).

## FUNDING INFORMATION

This project was supported by grants from the National Key R&D Program of China (No. 2021YFC2500500 to J.G. Wang); Hunan Provincial Natural Science Foundation of China [No. 2021JJ40852 to F. Luo, No. 2022JJ40675 J.F. Chen]; National Natural Science Foundation of China [No. 82100495 to F. Luo, No. 8220187 to J.F. Chen]; Scientific Research Project of Hunan Provincial Health Commission [No. 202203014009 to F. Luo, No. 202305037231 to J.F. Chen]; Scientific Research Launch Project for new employees of the Second Xiangya Hospital of Central South University (to F. Luo and J.F. Chen). China Postdoctoral Science Foundation (to F. Luo and J.F. Chen).

## CONFLICT OF INTEREST STATEMENT

Jingfei Chen, Qin Luo, Yingjie Su, Jiangang Wang, Zhenfei Fang and Fei Luo declare that they have no competing interests.

## CONSENT FOR PUBLICATION

Not applicable.

## Data Availability

Data used in this manuscript can be found at: https://wwwn.cdc.gov/nchs/nhanes.
